# Fair prioritization of casualties in disaster triage: a qualitative study

**DOI:** 10.1186/s12873-021-00515-2

**Published:** 2021-10-13

**Authors:** Vahid Ghanbari, Ali Ardalan, Armin Zareiyan, Amir Nejati, Dan Hanfling, Alireza Bagheri, Leili Rostamnia

**Affiliations:** 1 Emergency Nursing Department, School of Nursing and Midwifery, Kermanshah University of Medical Sciences, Kermanshah, Iran; 2 Health in Disaster and Emergencies Department, School of Public Health, Tehran University of Medical Sciences, Avecina Ave, Keshavarz Boulevard, Tehran, Iran; 3 Health in Disaster and Emergencies Department, School of Nursing, AJA University of Medical Sciences, Ehtemadzadeh st, West Fatemi St, Tehran, Iran; 4 Department of Emergency Medicine, School of Medicine, Tehran University of Medical Sciences, Tehran, Iran; 5 Clinical Professor of Emergency Medicine, George Washington University, Washington, DC USA; 6 Center for Medical Ethics and History of Medicine, Tehran University of Medical Sciences, Tehran, Iran; 7 Nursing Department, School of Nursing and Midwifery, Kermanshah University of Medical Sciences, Kermanshah, Iran

**Keywords:** Disaster, Triage, Priority setting, Medical ethics, Decision-making

## Abstract

**Background:**

Disasters may result in mass casualties and an imbalance between health care demands and supplies. This imbalance necessitates the prioritization of the victims based on the severity of their condition. Contributing factors and their effect on decision-making is a challenging issue in disaster triage. The present study seeks to address criteria for ethical decision-making in the prioritization of patients in disaster triage.

**Methods:**

This conventional content analysis study was conducted in 2017. Subjects were selected from among Iranian experts using purposeful and snowball sampling methods. Data were collected using semi-structured interviews and were analyzed by the content analysis.

**Results:**

Efficient and effective triage and priority-oriented triage were the main categories. These categories summarized a number of medical and nonmedical factors that should be considered in the prioritization of the victims in disaster triage.

**Conclusion:**

A combination of measures should be considered to maximize the benefits of the prioritization of causalities in disasters. None of these measures alone would suffice to explain all aspects of ethical decision-making in disaster triage. Further investigations are needed to elaborate on these criteria in decision-making.

**Supplementary Information:**

The online version contains supplementary material available at 10.1186/s12873-021-00515-2.

## Background

Mass casualties in a large-scale disaster can overwhelm the response capacity of any health care organization [1]. In such situations, available resources are inadequate and surge capacity strategies are significantly limited and not able to meet the needs of injured people [2–4]. Therefore, access to medical resources should be rationed. Triage, as a process of patient prioritization, enables health care providers to more effectively make use of scarce resources [2, 5].

Triage decision-making in disasters requires a different approach because of the scarcity of health care resources [5]. Disaster triage algorithms simply introduce primary principles on how to prioritize patients [6]. The previous experience of the Haiti earthquake and the SARS outbreak demonstrates how triage officers were uncertain about the accuracy of their decisions in patient prioritization [7–9]. Christian et al., to assess the effectiveness of a triage tool on patient outcome in pandemic situations showed that nearly 30% of triage officers lacked confidence in their decision-making and in approximately 55% of cases arbitration is needed [4]. Timbie, in a systematic review regarding strategies of physicians when allocating scare resources in mass casualty incidents and Goransso et al. who investigated the accuracy of nurses’ decisions in emergency triage revealed that physicians and nurses use different strategies in prioritizing injured people [10, 11]. Applying a different approach in decision-making threatens the consistency of process in disaster triage [4]. In order to reach consistency in patient prioritization, not only should clinical aspects be considered, but ethical aspects of decisions should also be considered [12]. Ethical guidelines lead to consistency, transparency, and accountability of decisions [7] and also prevent decision-making based on personal beliefs and feelings [13].

The greatest good for the greatest number is a utilitarian principle that has been accepted as the ethical base of triage in disaster situations [3, 14]. As this principle can be interpreted in several ways and the needs of each patient are of utmost importance in these situations, it is not clear how triage officers can apply the principles of triage that balance the need of each patient and the common good that supports the health-care services in disaster situations [14]. Furthermore, deciding on which patient should be prioritized is stressful and can lead to considerable ethical dilemmas [8, 15].

White believes that the principle of saving the most lives cannot lead to the best decisions in a disaster triage [16]. Furthermore, experiences of medical teams in the Haiti earthquake indicates that medical need was not the only prioritizing factor in such a situation [15]. Vetach showed that none of the utilitarian and egalitarian perspectives could guide ethical decision-making in triage [17].

Thompson states that due to the differences in ethical perspectives, each society must develop its own ethical guidelines based on cultural context and values for the prioritization of victims [12]. Due to the lack of appropriate guidelines on the ethical aspect of triage in Iran [13], this study aims to explore the criteria for ethical decision-making in patient prioritization in disaster triage.

## Method

### Research design

**Fig. 1:**
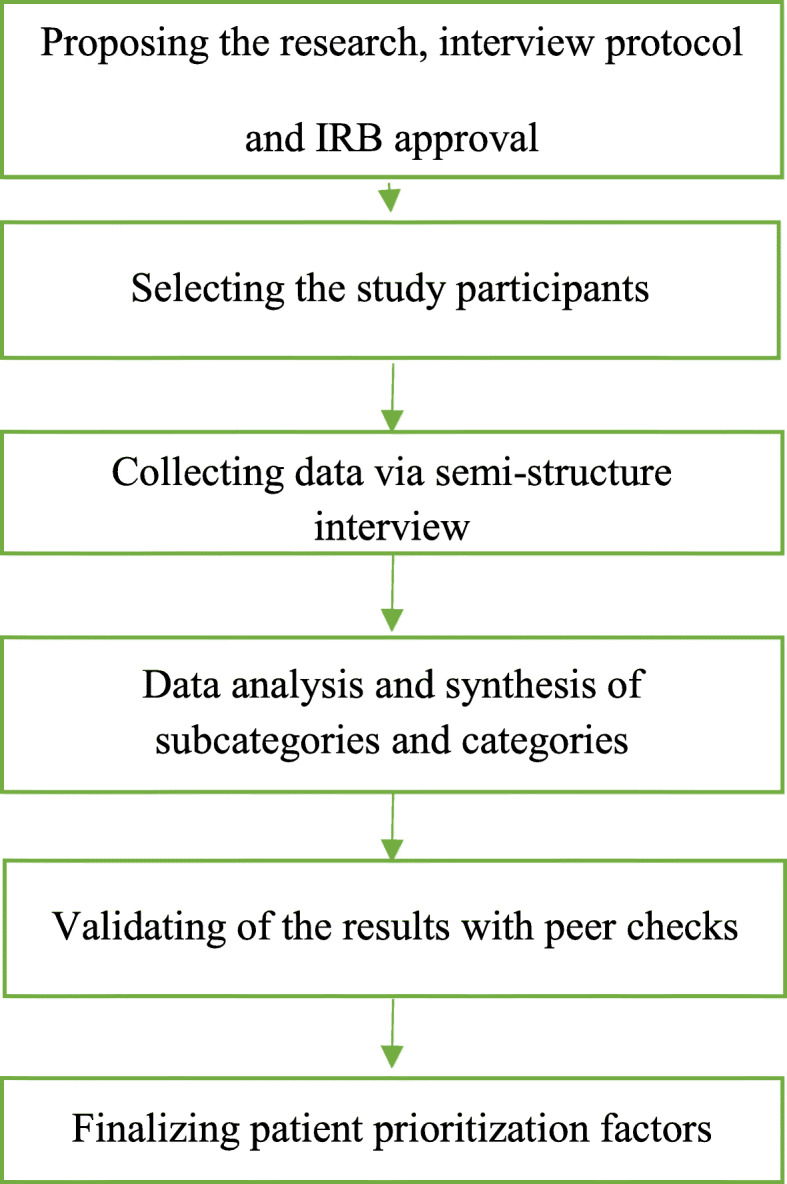
An Overview of key steps of research project

Descriptive qualitative research design was used. This method is one of the most feasible ways to explore the socio-cultural beliefs and values ​​of a society [18]. Figure 1 depicts the research and data analysis processes.

#### Ethical considerations

This study has been approved by the institutional review board of the School of Public Health of Tehran University of Medical Sciences (IR.TUMS.SPH.REC.1395.508). Participants were informed that participation in the study is voluntary and they could withdraw from the study at any time. Informed consent was obtained from all the participants. The IRB also confirmed that all methods were performed in accordance with relevant guidelines and regulations.

### Participants

Since disaster triage takes place in three levels including the pre-hospital setting, emergency department (ED) and intensive care unit (ICU), the participants were selected among pre-hospital and hospital clinicians [19]. The participants consisted of clinicians (Emergency Medical Technicians (EMT), hospital triage nurses, anesthetists, and physicians and nurses from the Iranian Red Crescent), and the inclusion criteria for the clinicians was that they should have lived experiences in performing triage in a disaster situation. As the aim of this study was to bring a comprehensive perspective about ethical decision-making in disaster triage, therefore, clerics and medical ethicists were also selected as non-clinician participants. These participants also had to be familiar with the subject of triage and patient prioritization in the health care system. Purposeful and snowball sampling methods were used to select the participants. In order to achieve maximum variation in perspectives, the participants were selected from different groups and cities (Tehran, Tabriz, Kerman, Shiraz, Qom, Mashhad, and Yazd).

### Data collection

Face to face, semi-structured interviews lasting between 30 and 75 min were conducted in the workplace and at a convenient time for the participants. The interviews were conducted by the first author (V.Gh) from April to October 2017. An interview guide was developed based on the purpose of the study, related literature and the research team experiences. The researcher (V.Gh) piloted the interview questions during the first two interviews with a triage nurse and an EMT. There were no modifications made in the interview guide and questions. Since there were no changes in the interview questions, the data collected in the pilot interviews were used in data analysis. At the beginning of the interview session, the interviewer explained the aim of the study. Then, participants were asked to describe one of their triage experiences. Subsequently, the interviewer asked them the following questions: 1) Did you experience any challenges in patient prioritization at the time of triage? 2) What were your challenges? As the topic was explored, questions progressed from general to specific to generate details. The major explorative questions of the interview were: 3) Did you face any type of moral challenge when prioritizing victims in disaster triage? 4) What criteria should be used for the prioritization of victims in disaster triage?. Probing questions were also asked to ensure the interviewer understood the participant’s responses and to provide an opportunity to obtain in-depth information.

After 24–25 interviews, no new code was generated from the interviews data, however, five more interviews were performed to ensure data saturation and for capturing data run-off.

### Data analysis

Data was analyzed using ‘content analysis’ was suggested by the Graneheim & Lundman proposed method [20]. Firstly, interviews were recorded and then transcribed verbatim, anonymously, and were reviewed several times to allow the first author to reach an overall understanding. To preserve participants’ privacy, all personal identifiers were transformed to a de-identified study number in the study files. Secondly, sentences or phrases that provided information about the ethical principles of patient prioritization in the triage were selected as semantic units. Thirdly, the semantic units were condensed, abstracted, and coded by V.Gh and L.R. Fourthly, codes were sorted into categories and subcategories based on constant comparison of their similarities and differences and consensus discussion by V.Gh, L.R, and A.Z. The separate categories coding sheets were then checked by the other authors for their further comments and then collaboratively interpreted and integrated. Finally, codes, subcategories, and categories were developed.

### Trustworthiness

Graneheim & Lundman proposed the credibility, dependability, and transferability of the data to ensure the trustworthiness of results in the content analysis studies [20]. Confirmability and credibility of data were enhanced by maximum variation of sampling, and prolonged engagement with data. Furthermore, members of the research team talked over the results to reach an agreement on the codes, categories, and subcategories. Peer checking was carried out by two other researchers who were not members of the research team to enhance the dependability of the results. In addition, to increase the transferability of the results, researchers tried to provide a clear and distinct description of sampling, data collection, data analysis, and reporting of the results.

## Results

Thirty interviews were conducted to gather the data in this study. Twenty-seven participants were male and three were female. The mean age of the participants was 40.3 (SD =10.7 years). The job experience of participants ranged from 3 to 17 years with a mean of 12.6 years (SD = 5 years). Twenty-one of the participants were clinicians (including six hospital triage nurses, four anesthetists, five Emergency Medical Technicians (EMT), and six physicians and nurses from the Iranian Red Crescent). Five medical ethicists and four clerics also participated.

Data analysis resulted in two main categories and nine subcategories. Table 1 shows categories and subcategories in the prioritization of casualties, which are explained as follows:

**Table 1:** An overview of categories and subcategories during data analysis

Category	Subcategory
**Efficient and effective triage**	Urgent needs
	Effectiveness of intervention
	The possibility of survivability
**Priority-oriented triage**	Special attention to the vulnerability of patients
	Prioritizing saving more years of life
	Prioritizing the productive life
	Prioritizing the social efficiency
	Prioritization based on possibility of service delivery
	Non-preferential prioritization

### Efficient and effective triage

The study participants expressed a number of points that should be considered in an ethical patient prioritization. According to explanations and examples are given by the participants, efficiency and effectiveness of the intervention is one of the main measures of decision-making in disaster triage. This is illustrated in the following sentence:

*“We prioritize patients who require immediate medical need, intervention would be more effective, and would offer victims a greater chance of survival.”* (P10, an anesthetist)

The efficient and effective triage category is comprised of three subcategories, including urgent needs, the effectiveness of the interventions and survivability.

#### Urgent need

All of the participants declared that an efficient triage prioritizes patients who require urgent emergency treatment and, if left untreated, would die. Regarding the extent of urgent medical treatment, clinicians put more emphasis on the status of vital signs and damages to vital organs. It is highlighted:

*"Our decision-making criteria are clear. As an example, we consider the level of blood pressure, consciousness and oxygen saturation, respiration and heart rate, and whether the patient needs mechanical ventilation or not, and then we choose who has the highest priority.”* (P12, an anesthetist).

Therefore, the vital signs, severity of injury, and damage to vital organs could be determinant factors for urgency or level of patients’ medical needs. It is emphasized:

*At first, patients are prioritized based on their vital signs and severity of damage* (P1, A triage nurse).

#### Effectiveness of the intervention

Nearly all of the participants believed that scarcity of resources arises in disaster situations. Therefore, in order for optimal utilization of resources, triage officers’ decisions should be as effective as possible. A number of participants stated that prioritizing patients based on the effectiveness of interventions is one of the ethical indicators of resource allocation in a disaster situation. It is highlighted by this comment:

*If we are going to assess our triage impact, we should look if I provide services to this patient does s/he benefit from our intervention or not.* (P 7, A triage nurse)

Furthermore, some of the participants believed that prioritization according to the effectiveness of interventions is an indicator of fairness in disaster triage*.* This is evidenced in the following quote:

*"In such situations, resources are scarce. So, it is completely fair to consider the effectiveness of our intervention. I mean in addition to the medical needs, we should also consider the effectiveness of the interventions".* (P27, a medical ethicist)

#### Possibility of survivability

More than half of the subjects believed that paying close attention to the possibility of patient survivability is another important factor in achieving the best results in disaster triage. Participants stated that triage officers have a responsibility to achieve the best possible outcome for the greatest number of injured. Therefore, when an injured person has a low chance of survival due to the severity of injury or comorbidities, it is best to place them in a lower level of priority. In addition, according to participants prioritizing such a patient leads to wastage of resources and futile care. The following example shows this:

*"If a victim is seriously injured, and nothing can be done to help them in the ICU or in the emergency department, these patients would not be my first priority."* (P19, an anesthetist).

### Priority-oriented triage

Most participants declared that, in addition to clinical criteria, a number of non-clinical criteria should be considered for patient prioritization. These non-clinical factors are patient vulnerability, saving more years of life, preserving a productive life, social efficacy, a possibility of service provision and non-preferential prioritization. This is highlighted by the following comment:

*In decision-making, we consider the patient age, whether the patient could be productive or could support a family or bring value to others in their community. (P15, an EMT)*

The participants whose opinions were categorized in this category believed that, by considering these factors, it enables the triage officers to establish a balance between utilitarianism and equality in decision-making, which are the components of ethical decision-making in triage.

#### Special attention to the vulnerable injured

Some participants expressed that, in the same situation, vulnerable groups (children, pregnant or lactating women, disabled people, and the elderly) should be prioritized as the afore-mentioned groups have less physical and psychological capabilities.

Moreover, most of the participants from the Red Crescent staff and EMTs expressed that communities are more emotional towards the care of these individuals especially in the pre-hospital field. The participants emphasized that special attention should be paid to women, children, and other vulnerable groups during the triage. This item is highlighted in the following sentences:

*"When I was triaging, I unintentionally paid more attention to children, pregnant or lactating women, and disabled people."* (P8 a nurse working in the Red Crescent).

#### Prioritizing saving more years of life

More than half of the participants believed that, although saving more lives is the main principle of disaster triage, in patients with similar medical needs, age could be a reasonable criterion for patient prioritization. According to a number of participants, children and adolescents have a greater chance of recovery and improvement; so, triage officers should prioritize children. The following sentences highlight this:

*"In my opinion, age is important in the triage of casualties. It is reasonable to be more hopeful regarding children, and it is always in your mind that, children are just beginning their lives and have a greater chance of survival."* (P13, a physician working in the Red Crescent).

#### Prioritizing a productive life

Based on a number of participants’ narrations, lifesaving is not the only aim of disaster triage, triage officers must also, consider saving those who have the opportunity of living with a better quality of life. In general, some of the participants believed that a triage officer should try to save patients who would be able to live independently and help the affected community in some way in the future. The following quote demonstrated:

*"It may be concluded that a certain patient would not be able to live independently even after all that was done for them. Consequently, a lower priority is given to this patient in comparison to patients who are more likely to recover and be able to lead an independent life."* (P21, an anesthetist)

#### Prioritizing the social efficiency

Some of the participants presented several examples, which indicated that the worth of an individual within the family and society should be considered for patient prioritization. Some of the participants claimed that by saving a parent, the family could maintain its independence and function after a disaster. The following sentences express this idea:

*“You may be faced making a decision to prioritize a man who is parenting 5 or 6 children or a single man; surely you would give priority to the casualty who is parenting children.”* (P22, an anesthetist)

Moreover, participants stated that if a person could go on to help other victims in a disaster due to their role, knowledge, or expertise, these casualties should be given priority. Participants believed that these individuals could help to maintain community function in a disaster situation. Then, those who responded to the injured in a disaster should be put in the higher priority. It is clarified in this quote:

*"If one of the member of the response team is injured during the response to victims, it is ethically acceptable that these injured be prioritized to receive services."* (P7, a medical ethicist).

However, a number of participants expressed that this should not lead to special privileges for these groups.

#### Prioritization based on the availability of service

Nearly half of the participants agreed that during prioritization of patients, the triage officers should not only consider the severity of the individual needs but should also pay attention to the type and amount of resources needed by the victims. From the participants’ perspective if only the medical needs of victims are considered in triage, the person who is given priority may die because of the severity of damage. Therefore, the resources allocated to this person would be wasted. This is highlighted in the following sentences:

*“For example, you work in a city where you do not have access to a neurosurgeon. Well, if you prioritize a victim who needs neurosurgery, you have not helped them because there is no neurosurgeon there.” (P1 a hospital triage nurse).*

#### Non-preferential prioritization

A number of participants believed that when several patients’ are in the same priority level or triage category, prioritization based on a first come, first served or lottery is the last option. Participants expressed that applying this factor could prevent the contribution of other factors such as recommendation, personal feelings, and emotions of the triage officers being involved in decision-making. The following quote emphasizes this point:

*"If you have ten casualties that all of them are in the same priority level, you can prioritize them by lot. We have a rule in Islam that says drawing lots is a solution for all difficult situations to make a decision.”* (P23, A cleric)

## Discussion

This study provided the opportunity to explore the perspective of Iranian health professionals on factors contributing to ethical decision-making in patient prioritization during disaster triage. Since triage is a response to an imbalance between health care needs and available resources in a disaster situation [21], triage officers should try to prioritize patients in a way that provides the greatest good for the greatest number of people. As a result, the effectiveness and the efficiency of services should be a measure for patient prioritization during disaster triage [22].

As triage seeks to save people whose lives are at risk and its aim is to direct resources to the injured people who have greater medical needs [21]. Thus, patient prioritization based on medical needs will decrease the morbidity and mortality among the injured [23]. Then, it seems that considering patient medical needs as a prioritizing factor leads to maximum use of available resources in disaster situations.

Allocating resources to patients who have a greater chance to benefit from the resources is another goal of disaster triage [4]. Thus, it is fair to prioritize casualties who could benefit from the resources [24]. According to Lin and Anderson-Shaw, the effectiveness of interventions in patient prioritization can preserve resources; as a result, a larger number of victims can receive care [25]. This finding is consistent with Caro et al. and Kuschner et al. [2, 24], which indicated both patient needs and the chance to benefit from the allocated resources should be considered simultaneously in patient prioritization.

Our results also showed that prioritization of patients based on their chance of survival was another measure that triage officers should consider in disaster triage. Generally, triage is more effective when it prioritizes patients who are more likely to survive. Moreover, giving priority to patients who have little chance to survive is a suboptimal use of scarce resources in a disaster [4]. Kuschner et al. mentioned that survivability is one of the factors that should be considered during patient prioritization [2].

A number of participants thought considering just medical needs and the effectiveness of interventions as prioritizing measures cannot lead to the common good in disaster triage. Therefore, they suggested triage officers should consider other factors like protecting vulnerable groups, prioritizing young people or parents in disaster triage.

Interestingly, a number of participants believed that in the same medical condition, vulnerable groups should be prioritized. Barrnett et al. argued that resource allocation in disasters should be sensitive to the needs of vulnerable groups [26]. Vawter et al. believe that, vulnerable groups should have the same chance in decision-making, and that barriers to service provision to these people should be resolved [22].

The results showed that saving more years of life should be another prioritizing factor in disaster triage. White et al. argued that saving more years of life provides a more complete explanation for the greatest good for the greatest number rather than saving a larger number of lives [16]. Participants’ opinions suggested the prioritizing of younger people provides greater justification for the equal chance of individuals to pass all stages of life. In contrast, Levin et al. stated that since saving the life of an elderly person or a young person is of equal importance, age should be considered along with other factors [1].

The importance of independence and efficiency of the injured as a prioritization factor is highlighted in our findings. Based on the maximum principles, allocation of health care resources to patients who will achieve the best outcome are essential to maintain equity among patients [27]. As physical disability leads to long-term dependency after disasters [28], it seems the independence and efficiency of a casualty as an outcome, could be another measure for patient prioritization. Kuscher et al. have also mentioned that promoting and maintaining the quality of life should be considered in the triage process [2]. However, other studies disagreed with considering the quality of life as a prioritization factor. These researchers believed that this measure is a subjective criterion and may discriminate against people with disabilities and chronic diseases [29, 30].

Our results also suggest that some Iranian professionals place importance on the prioritization of victims regarding their social efficiency. This finding seems to be well supported by the wellness of society as a whole. It is acceptable that individuals who can be more effective be given higher priority [31]. Smith believes health professionals, public safety officers, and government decision-makers should be prioritized in disaster triage [32]. However, other studies oppose the prioritization of casualties based on their social value or prioritization of healthcare workers [3, 16, 33] because they believe the life of each victim has equal value and it is not acceptable to mention this factor as a prioritizing measure. Therefore, further investigations are needed to establish how social efficacy should be considered in patients’ prioritization.

Some participants indicated that having parental responsibility also could be a factor in patient prioritization in disaster triage. This finding is particularly important and it could be explained the socio-culturally context of this research. Because Khankeh et al. reported that the loss of one of the parents after a disaster results in the disintegration of the family [28]. Family Life Management has been introduced as one of the most effective cultural recovery factors after natural disasters in Iran [34]. It has also been suggested that family reunification should be considered as one of the components of a disaster recovery plan [28]. Therefore, it seems that considering this factor in patient prioritization could lead to a better and quicker recovery after a disaster. Silva et al. also suggested that having parental responsibility and caring for others could be a factor in patient prioritization [35]. This finding highlights the prominent role of the family from Iranian professional perspectives. This important issue for future study.

The analysis showed that the importance of the availability of required services for patient prioritization. Although prioritizing the injured merely based on the triage level is simple, the scarcity of resources at the time of a disaster is not taken into account in this prioritization method. Therefore, in some cases, patient prioritization should be based on the available resources relative to the patient’s medical needs [5]. This finding is in line with Sprung et al.’s study, which revealed the lack of required expertise, and resources could lead to the removal of patients from the ICU waiting list in a pandemic [36].

The last finding of this interview study was the non-preferential prioritization of victims in disaster triage. Taurek argued that as all humans’ life equally valuable, so in a scarce resource situation, triage officers ought to give an equal chance to each individual casualty [37]. In other words, first-come, first-served or random selection would give an equal chance to all salvageable patients [21]. It seems by considering this measure, resources would be distributed fairly among all casualties. Matheny Antommaria et al. believed that when resources are limited, prioritization of the injured should be based on a lottery [38]. Silva et al. considered queuing as a practical measure for prioritizing patients during a pandemic [35]. However, other studies opposed the selection of those injured based on a first come, first served [16, 22]. Vawter et al. suggested that patient prioritization based on this measure is incompatible with the goals of public health in disasters [22].

### Limitation

Although this is a qualitative study based on Iranian experts, the result can be generalized to similar cultural contexts. As is well known, generalizing from qualitative research is based on analytical and inferential not statistical-probabilistic generalizability [39].

## Conclusion

The results of the study highlight a pluralistic perspective (medical need, efficiency, and effectiveness of medical interventions, the productivity of saved injured in family and community, and saving vulnerable groups and resources) in decision-making for patients’ prioritization. A combination of these factors is needed to make an ethical decision in disaster triage. None of these criteria alone can provide a complete explanation for fair patient prioritization in disaster triage. Further studies are needed to determine the importance of each of these factors in patient prioritization. It is important to evaluate the application of these principles to achieve the greatest good for the greatest number in disaster triage. This study will be useful to triage officers and emergency nurses in relation to what constitutes an ethical decision for patient prioritization in disaster triage.

## Supplementary Information

**Additional file 1.**

## Data Availability

The datasets generated and/or analyzed during the current study are not publicly available due to participant confidentiality but are available from the corresponding author on reasonable request.

## Abbreviations

PP: Patient Prioritization
EMT: Emergency Medical Technicians
ICU: Intensive Care Unit
