# Supplementary material for: Fair prioritization of casualties in disaster triage: a qualitative study
Source: BMC Emerg Med. 2021 Oct 13;21:119. doi: 10.1186/s12873-021-00515-2 (PMC8513386; doi:10.1186/s12873-021-00515-2)
Supplement: Supplementary file 1 — Additional file 1. [file 12873_2021_515_MOESM1_ESM.docx]

Please introduce yourself and explain your experience in doing triage in time of a disaster.

1) When you had done triage, Did you experience any challenges in patient prioritization?

2) What were your challenges?

3) Did you face any type of moral challenge when prioritizing victims in disaster triage?

4) What criteria should be used for the prioritization of victims in disaster triage?
